# High-throughput investigation of crystal-to-glass transformation of Ti–Ni–Cu ternary alloy

**DOI:** 10.1038/s41598-019-56129-z

**Published:** 2019-12-27

**Authors:** Jian Hui, Haiqian Ma, Zheyu Wu, Zhan Zhang, Yang Ren, Hengrui Zhang, Lanting Zhang, Hong Wang

**Affiliations:** 1 Materials Genome Initiative Center, Shanghai Jiao Tong University, Shanghai, 200240 China; 2 School of Materials Science and Engineering, Shanghai Jiao Tong University, Shanghai, 200240 China; 3 Zhiyuan College, Shanghai Jiao Tong University, Shanghai, 200240 China; 4 X-ray Science Division, Advanced Photon Source, Argonne National Laboratory, Argonne, Illinois 60439 United States

**Keywords:** Metals and alloys, Synthesis and processing

## Abstract

A high-throughput investigation of metallic glass formation via solid-state reaction was reported in this paper. Combinatorial multilayered thin-film chips covering the entire Ti–Ni–Cu ternary system were prepared using ion beam sputtering technique. Microbeam synchrotron X-ray diffraction (XRD) and X-ray fluorescence (XRF) measurements were conducted, with 1,325 data points collected from each chip, to map out the composition and the phase constitution before and after annealing at 373 K for 110 hours. The composition dependence of the crystal-to-glass transition by solid-state reaction was surveyed using this approach. The resulting composition–phase map is consistent with previously reported results. Time-of-flight secondary ion mass spectroscopy (ToF-SIMS) was performed on the representative compositions to determine the inter-diffusion between layers, the result shows that the diffusion of Ti is the key factor for the crystal-to-glass transition. In addition, both layer thickness and layer sequence play important roles as well. This work demonstrates that combinatorial chip technique is an efficient way for systematic and rapid study of crystal-to-glass transition for multi-component alloy systems.

## Introduction

Metallic glasses (MGs) are metal alloys that lack long-range order or periodicity. They often possess some exceptional characteristics that are unattainable from crystalline alloys, such as outstanding mechanical properties^1–3^ and processability^4^. The first metallic glass was prepared by Duwez and co-workers^5^ in the 1960s via a rapid quenching technique at a cooling rate of 10^5^–10^6^K/s. However, such a fast cooling rate is very difficult to achieve in industrial production. And at the same time, because of the limited ability for the alloy to form amorphous structure, only a thin amorphous strip of limited compositions can be prepared. Attempts have been made to develop alternative methods to form amorphous alloys. In the 1980s, a new category of amorphization techniques based on solid-state reactions was developed, such as mechanical alloying^6^, ion beam mixing^7^, hydrogen absorption^8^, and interlayer diffusion^9,10^, to transform crystalline precursors of powder or thin film into metallic glasses.

In the interlayer diffusion method, a stack of multiple layers of different compositions is deposited. The solid-state reaction occurs in two steps^11,12^, interdiffusion of the reactants and nucleation/crystallization of end products, and it normally results in crystalline materials. However, if one component of the alloy diffuses much faster than the other components, the crystallization of the new structure is hindered^13^, and thus a crystal-to-glass transition occurs. The interlayer diffusion method is of great importance from both fundamental science and engineering application^14^ standpoints.

Several factors were found to affect the amorphization ability of a multilayer system, including the composition of reactants^15^, the layer thickness and sequence^16,17^, and the state of the precursor^13^. Traditionally, MGs composition screening of this kind has to go through a series of trial-and-error experiments^18–20^. Such an approach is both labour-intensive and time-consuming, so it is not efficient for exploring a wide compositional range.

Combinatorial multilayered thin film synthesis technique provides advantage in studying the interlayer diffusion, with which a vast number of multi-layer stacks are fabricated in parallel on one substrate by varying the thicknesses of each component deposited at different positions^12,21–29^. Combined with high-throughput characterization, the screening and optimization processes of MGs can be dramatically accelerated.

In this paper, we report a systematic investigation of amorphous alloy formation in the Ti–Ni–Cu ternary system via a combinatorial approach. The system is well known to have an amorphous composition region and a fast diffuser Ti^30–32^. The ternary thin-film combinatorial library was fabricated using a sequential multilayered deposition system. High-throughput structural and compositional characterizations were conducted on a synchrotron source and automatic data analysis based on machine learning was employed to quickly generate knowledge of the phase constitution and phase-evolution diagram in the solid-state reaction. Our results demonstrated a high-throughput strategy to systematic and rapid study of crystal-to-glass transition for ternary alloy systems.

## Results and Discussion

### Multilayered thin-film alloy deposition

**Figure 1:**
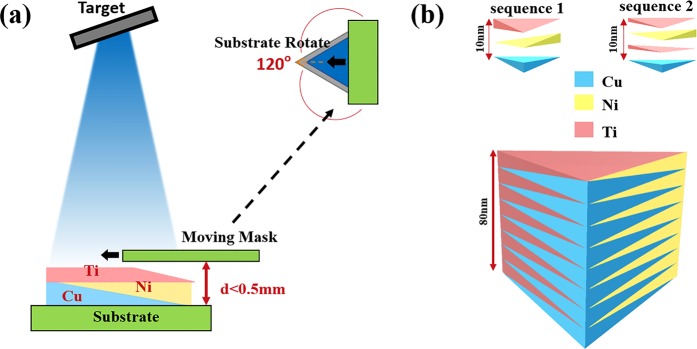
(**a**) Schematic illustration of the deposition procedure with a moving mask. (**b**) Cross-section of different layering sequences of multilayered film with gradient composition spread.

As illustrated in Fig. 1a, the combinatorial thin-film precursor covering the entire composition range of Ti–Ni–Cu ternary system, were deposited following a procedure described by Xing *et al*.^33^ on quartz glass substrates (25.4 mm × 25.4 mm × 2 mm) using a custom-designed high-throughput combinatorial ion beam deposition system (HTC-IBD) at room temperature with a base vacuum pressure of 1 × 10^−6^ torr. The ternary thin-film precursor consisted of a sequentially deposited stack of wedge-shaped layers, that were created by a mask moving linearly across the substrate during deposition. The coated area is triangular-shaped (20 mm in side length) with a total thickness of 80 nm across. The stack was divided into 8 cycles of 10 nm layer groups. Two different layer sequences were used: a three-layer sequence of Cu → Ni → Ti (sequence 1) and a four-layer sequence of Cu → Ti → Ni → Ti (sequence 2), as shown in Fig. 1b. Then the as-deposited precursors were sealed in evacuated quartz glass tubes and placed in a vacuum oven for heat treatment. One of the key factors in the solid-state amorphization is to ensure the annealing temperature is lower than the formation temperature of intermediate compound. Therefore, a low annealing temperature (373 K) was selected so that the nucleation and growth of the crystalline phases were suppressed.

### Phase identification in Ti–Ni–Cu libraries

**Figure 2:**
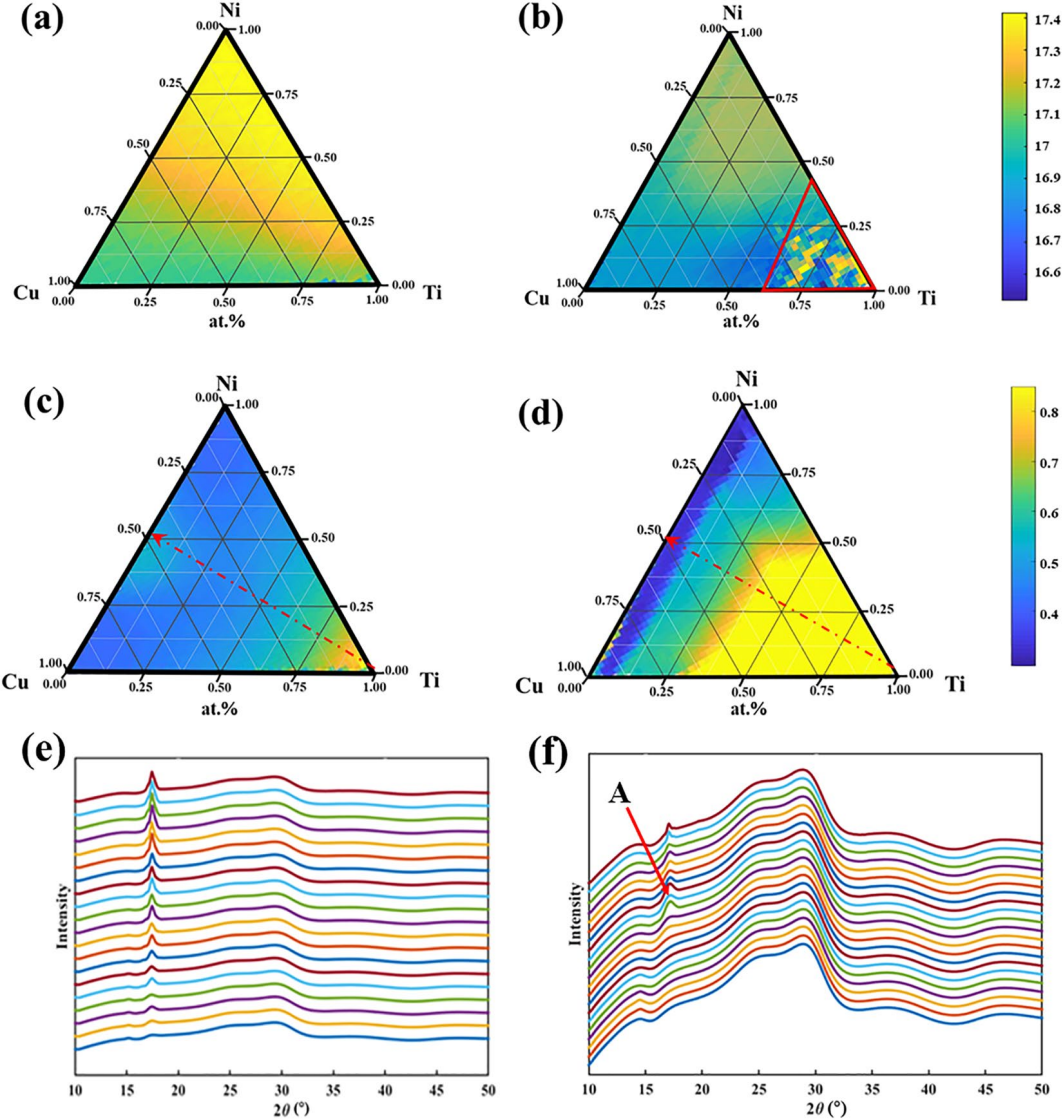
Mapping of peak position measured in high-throughput XRD experiments (**a**) as-deposited, (**b**) annealed at 373 K for 110 h, (**c**) the mapping of FWHM(sigma) of the peak as-deposited, (**d**) the mapping of FWHM of the peak annealed at 373 K for 110 h, (**e**) the XRD pattern following the red arrow at as-deposited state and (**f**) the XRD pattern of annealed at 373 K for 110 h (**A** is chosen to be the boundary between amorphous and crystalline structure).

High-throughput micro-beam XRD/XRF measurements were performed simultaneously to map out the structure and composition of the combinatorial material chips of Ti–Ni–Cu deposited with **sequence 1** before and after annealing at 373 K for 110 hours. The map of the peak position of the Ti–Ni–Cu ternary system extracted from high-throughput experimental observations using automatic data processing is plotted in Fig. 2a,b. Across the whole chip, there is only one prominent peak located between 16–19 degrees. The XRD spectrum of the quartz glass displays a broad hump. In the as-deposited state, the peak distribution of the whole chip is primarily related to the components of Ni and Cu, indicating that the crystalline peaks of the samples come from Ni and Cu. The peak position in the Ni-rich region is close to the (111) fcc-Ni peak, and that in Cu-rich region is close to the (111) fcc-Cu peak. A significant change occurred after heat treatment; the peak position of the entire sample was shifted to a lower angle. The peak in the red triangle region near Ti corner was almost invisible after annealing at 373 K for 110 h. Therefore, the peak position in this region cannot be clearly determined.

The map of the full width at half maximum (FWHM) of the Ti–Ni–Cu ternary system is shown in Fig. 2c,d. The FWHM decreases following the red arrow with the contour lines almost parallel to the Cu–Ni edge. After heat treatment, the FWHM increased significantly compared with as-deposited state up to about 10% Ti boundary, while the FWHM at less than 10% Ti content is reduced. The diffraction pattern following the red arrow line before and after heat treatment are illustrated in Fig. 2e,f. The intensity of the peak significantly increased with the decreasing composition of Ti in the as-deposited state. After heat treatment, the peak strength weakened, and the peak width increased in most areas except for along the edge of Cu–Ni. The spectra from the corner of Ti to the boundary of about 50% Ti displayed no apparent peaks, which is indicative of the transition of crystal to glass during heat treatment. Although the peak strength of the Cu–Ni edge decreased significantly, the width of the peak became narrower. Before and after the solid-state reaction of the Ti–Ni–Cu ternary system, there were two regions showing the greatest change. In Ti-rich regions, the transition from crystal to the amorphous structure was completed, while in the region with less than 10% Ti content, the degree of crystallization was higher than it was before heat treatment.

Fister *et al*.^34^ pointed out that the thickness of the reacting layer affects the final product of the solid-state reaction, and the smaller the film thickness, the higher the nucleation temperature is required. Meng *et al*.^35^ studied the interdiffusion reaction in Ti/Ni system at 523 K for 10 h with an individual layer thickness of 10 nm (the overall atomic ratio is Ti_44_Ni_56_). They found that the Ti/Ni reaction product was a simple intermetallic. However, in this study, the peak strength weakened, and the peak width increased distinctly, the layered sample tends to form an amorphous structure instead after heat treatment at 373 K for 110 h with an individual layer thickness around 5 nm as shown in Fig. 2c,d. According to the Ti–Ni binary phase diagram^36^, the same intermetallic compounds form at both temperatures. Therefore, it is the layer thickness that affects the state of the final product of the reaction in this case. It is beneficial to the formation of the amorphous structure to decrease the thickness of the layer. We believe that the amorphous region will become larger when the thickness of the reacting layer is further reduced, and the reaction time is increased.

**Figure 3:**
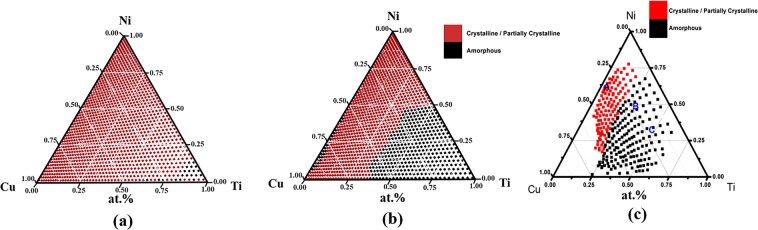
The maps of phase category of the experimental data (**a**) as-deposited, (**b**) after annealing at 373 K for 110 h and (**c**) the recently reported data of the Ti–Ni–Cu thin film deposited by co-sputtering^30^.

We used the FWHM of the X-ray diffraction peak as a parameter to classify whether a point on the combinatorial chip was amorphous or crystalline. As shown in Fig. 2e,f, the FWHM changed continuously, a criterion is necessary to make the determination. The XRD spectra in Fig. 2f shows a clear transition from amorphous to crystalline state. The FWHM at the transition (the broadest peak in the group, marked as A) was designated as a criterion for amorphous phase. The resulting categories (amorphous/crystalline and partial crystalline) before and after heat treatment were plotted in Fig. 3a,b. There is a small amorphous region near the corner of pure Ti in the as-deposited state. The amorphous region expands after heat treatment from the corner of Ti to a boundary at about 30% of Ti content. The results of the category are in good agreement with the data reported by Ludwig, as illustrated in Fig. 3c^30^, except that their data did not cover the entire ternary phase diagram. The boundary of the amorphous region in the Ni-rich side is 50% Ti, and the boundary of the Cu-rich side can be extended to about 30% Ti. In comparison, the amorphous/crystalline boundary reported by Ludwig was parallel to the edge of Ni–Cu at about 25% Ti content. The differences in the boundaries may be due to the difference in the synthesis method and the criteria for amorphous phase determination. The present results are generally in agreement with the diagram by rapid quenching method^37^ except that the Ti-rich corner shows crystalline state instead of amorphous state. This is due to the different amorphous formation mechanisms of the two methods.

### Interdiffusion in Ti–Ni–Cu multilayer films

**Figure 4:**
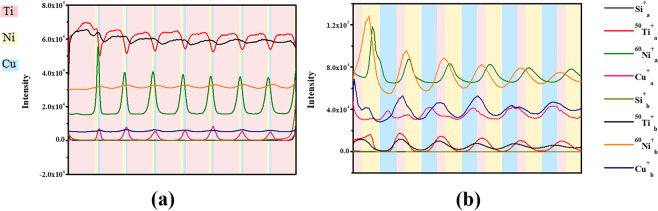
ToF-SIMS elemental depth profiles of Ti_75_Ni_15_Cu_10_ (**a**) as-deposited (using **a** as the index) and after heat treatment at 373 K for 110 h (using **b** as the index) and Ti_10_Ni_50_Cu_40_ (**b**) as-deposited and after heat treatment at 373 K for 110 h (different colour of the background represents different element layers).

The degree of the interdiffusion was characterized by ToF-SIMS. Two areas with overall compositions of Ti_75_Ni_15_Cu_10_ and Ti_10_Ni_50_Cu_40_ from the chip deposited as sequence 1 (Fig. 1b) were analysed, representing the Ti corner and the area with less than 10% Ti content, respectively. The depth profile of the elements Ti, Ni, Cu, and Si are displayed in Fig. 4. Si was used to mark the boundary between the sample and the substrate. The ^50^Ti^+^and ^60^Ni^+^ signals were chosen to represent the distribution of Ti and Ni, respectively, since the intensity of Ti^+^ and Ni^+^ were sometimes beyond the upper detection limit. For both samples, the distribution of each element before heat treatment was highly consistent with the designed layer stack. We noticed that the Ni peaks shifted slightly towards the Ti layer during deposition, which was mainly because of the large negative heat of mixing^38^ between Ni and Ti. During the deposition process, the deposition temperature and the large negative enthalpy of mixing promoted the inter-diffusion of Ti and Ni. In comparison, the peak position of Cu was unchanged.

After heat treatment, the Ti, Ni, and Cu peak heights were all reduced to a large extent, as shown in Fig. 4a, suggesting that a significant amount of interdiffusion has occurred in the Ti_75_Ni_15_Cu_10_ multilayered thin film. The range of Ti ions was reduced, confirming the distinct diffusion of Ti in the reacting layers. The distribution of Ni and Cu was almost flat, indicating that they are well mixed during annealing. In the meantime, Ti diffused rapidly and passed through the Ti/Ni and Ni/Cu interfaces. The Ni peak was slightly leaning to the Cu layer, while the peak position of Cu was almost unchanged. In Fig. 4b, the Ti peak in the Ti_10_Ni_50_Cu_40_ multilayered film was broadened and shifted towards the Cu layer after heat treatment. Overall, the distribution of Ti changed the most in both multilayered thin films, as a result of the fast diffusion in the solid-state reaction. The peak positions of Ni and Cu both moved to the Ti/Ni and Ti/Cu interface. Unlike Fig. 4a, the amplitude of the Ni and Cu curves became larger. Therefore, under the current experimental condition, the stack was not completely mixed in the depth direction.

**Figure 5:**
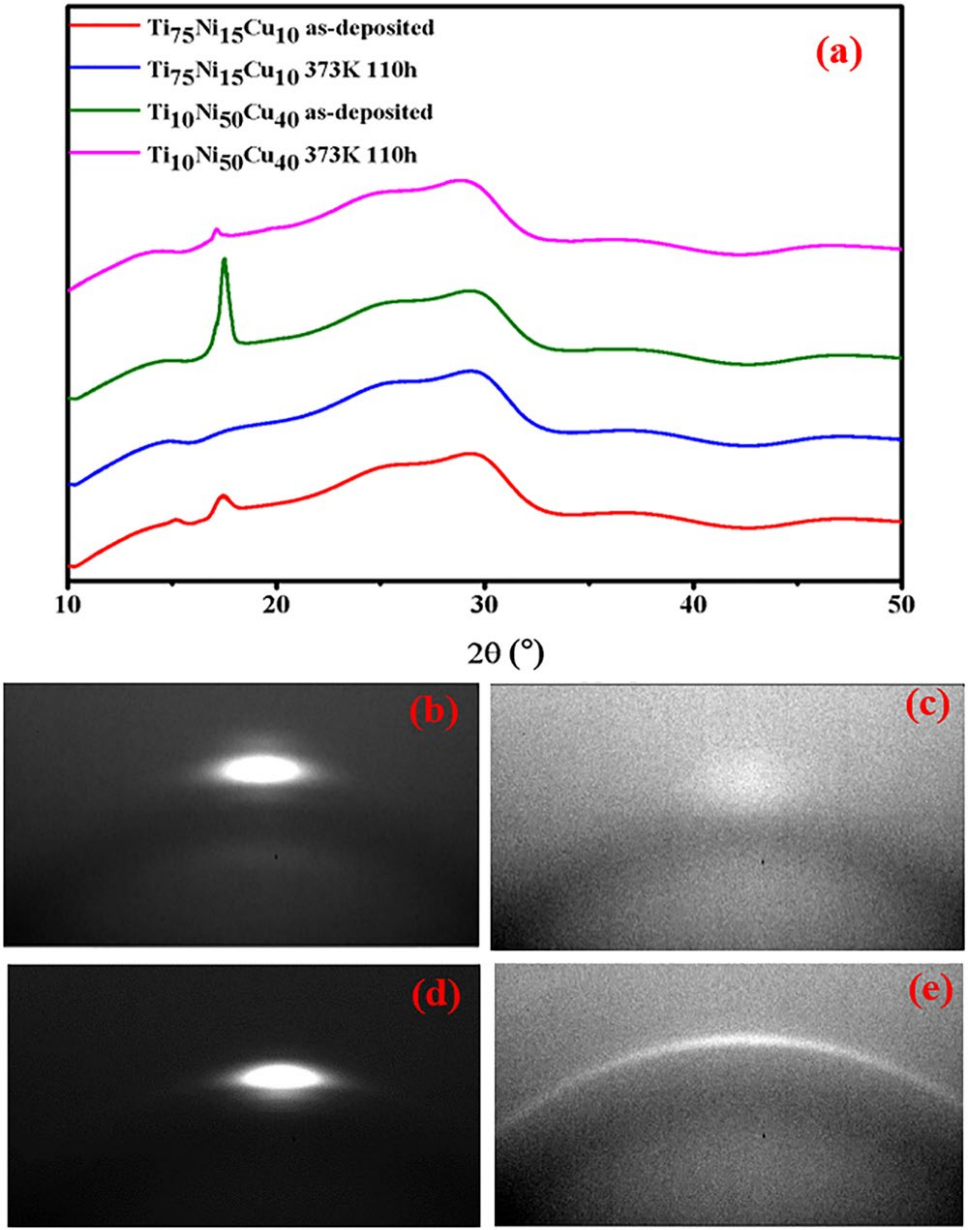
2D Synchrotron XRD pattern of Ti_75_Ni_15_Cu_10_ and Ti_10_Ni_50_Cu_40_ (**a**) XRD analysis data, (**b**) as-deposited Ti_75_Ni_15_Cu_10_ and (**c**) after annealing, (**d**) as-deposited of Ti_10_Ni_50_Cu_40_ and (**e**) after annealing.

The synchrotron X-ray diffraction patterns for the Ti_75_Ni_15_Cu_10_ and Ti_10_Ni_50_Cu_40_ multilayered thin film before and after annealing are given in Fig. 5. The crystalline peak disappeared completely with annealing in Ti_75_Ni_15_Cu_10_, indicating that the amorphous phase was formed before any crystalline phase nucleated. In contrast, the peak of Ti_10_Ni_50_Cu_40_ became sharper even though the height was decreased. As shown in Fig. 5d,e, the as-deposited precursors with strong orientation became randomly oriented polycrystalline during annealing. Thus, the initial stronger diffraction spot was spread out to a complete ring, resulting in narrower but weaker signals received by the detector. According to the ICSD database, Ti_10_Ni_50_Cu_40_ is likely to crystallize into intermetallic compounds of Ti_3_Ni_4_ (No. 105422) and Ti_3_Cu_4_ (No. 103134) during the solid-state reaction at this temperature.

**Figure 6:**
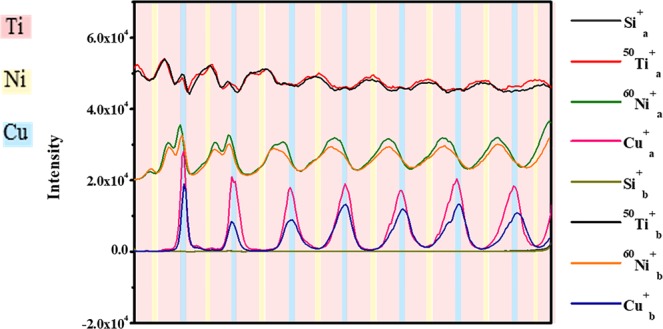
ToF-SIMS elemental depth profiles of Ti_72_Ni_13_Cu_15_ designed as sequence 2, as-deposited (labeled as **a**) and after heat treatment at 373 K for 110 h (labeled as **b**).

In an attempt to further decrease the diffusion distance of Ti, four-layer cycle stacks (Fig. 1b, sequence 2) were analysed. In the ToF-SIMS depth profile in Fig. 6, the peaks from two of Ti layers merged into one at the designed Ni location even in the as-deposited state. In the meantime, the Ni peak shifted toward Cu and the position of Ni and Ti were staggered. The Cu peak remained unchanged. Again, mixing between Ti and Ni occurred during deposition.

After heat treatment, the Ti curve became smoother, as did the Cu and Ni curves. Furthermore, the Cu and Ni peaks shifted slightly towards the Ti layer. The migration directions of Cu and Ni are random. However, the profiles are not significantly changed. The results indicated that the Ti/Cu interface appeared to serve as a barrier to block interdiffusion. Therefore, the layering sequence is another critical parameter that has a strong impact on the solid-state reaction in addition to the layer thickness.

## Conclusion

In this paper, we have demonstrated a new high-throughput approach enabling systematic and rapid study of the crystal-to-glass transformation of ternary alloys via solid-state reaction. The Ti–Ni–Cu crystal-to-glass transition map covering the entire ternary composition space was generated by high-throughput synchrotron XRD and XRF characterizations on a single combinatorial thin-film chip. The glass-forming composition agreed well with the literature. ToF-SIMS study showed that the Ti diffusion is the key factor for the crystal-to-glass transition, while the thickness of the Ti layer and the layer sequence of the component are also important in affecting the final product of the solid-state reaction.

## Methods

### XRD and XRF measurements

The structure and composition of the chips were measured by microbeam XRD and XRF at a high-throughput X-ray set up on the Sector 33-ID-D of the Advanced Photon Source, Argonne National Laboratory, USA. The X-ray photon energy was tuned to 20 keV (λ = 0.062 nm). At an incident angle of 10 degrees, the beam was focused on the sample to a size of about 75 × 100 µm^2^ by a pair of KB mirrors. A Newport 6-circle diffractometer was used to hold the sample and the detector. The chip was scanned with a step size of 360 µm in the longitudinal direction and 400 µm in the lateral direction with a total of 1,325 diffraction patterns recorded from the triangular region on each combinatorial chip. A Pilatus II-100k detector was mounted on the Del arm of the Newport at about 300 mm from the sample. For every spot on the sample, the diffraction signal was collected through a trajectory scan of Del’s arm from 10 to 50 degrees, which covered 2-theta from 7 to 57 degrees.

A fluorescence detector (KETEK, AXAS-D, GmbH) was installed at about 20 mm away from the sample, perpendicular to the diffraction circle to collect X-ray fluorescence data simultaneously with XRD measurement. The results were calibrated on a few selected regions of the combinatorial libraries by wavelength dispersive X-ray spectroscopy (WDS) in an electron-probe microanalyzer (EPMA, JEOL, JXA-8530f Plus). The XRF data was fed into an in-house model to correct any offset of the composition coordinates due to experimental errors.

### XRD and XRF data processing

The two-dimensional (2D) diffraction files taken on each spot were reduced to one intensity vs. angle diffraction spectrum (see Fig. 2), using a modified RSM3d software package code provided by the beamline. The background of each diffraction spectrum was removed by subtracting the diffraction spectrum of a blank substrate obtained in an identical way. The region between 16–19 degrees was further analysed by fitting the curve to a combination of one Gaussian peak, one Lorentzian peak, and a linear background. The Gaussian peak width (Sigma) and peak position were taken and plotted out as a function of the sample x & y position. Mapping the phase diagram process, it is necessary to introduce point-by-point XRF data to determine the coordinates of each point on the chip and map the components on the phase diagram coordinate.

**Figure 7:**
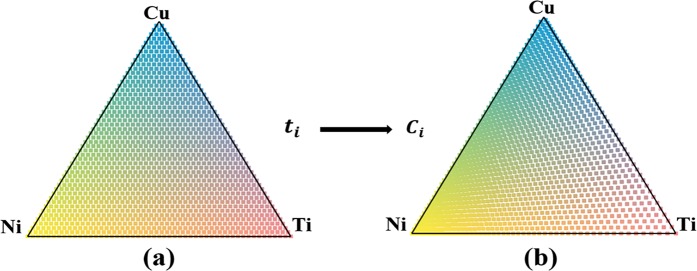
The diagram of Ti–Ni–Cu combinatorial library with 1,325 measurement points is illustrated (**a**) in spatial coordinates and (**b**) in composition coordinates. Each dot represents a measurement point.

The composition of *c*_*i*_ on the combinatorial chip was determined by the thickness of each component, *t*_*i*_, as described by

$${c}_{i}=\frac{{t}_{i}{\rho }_{i}/{Z}_{i}}{{\sum }_{i}{t}_{i}{\rho }_{i}/{Z}_{i}},\,{\rm{and}}\,{\sum }_{i}{t}_{i}={\rm{const}}$$

where *Z*_*i*_ is the atomic weight and *ρ*_*i*_ is the density of component *i*. For the Ti–Ni–Cu ternary system, since *t*_*i*_ changes linearly along the direction of the mask movement, while *ρ*_*i*_/*Z*_*i*_ varies with the element, the compositional coordinates are not linear to the spatial coordinates. Thus, the evenly distributed measurement data points are most concentrated around the corner of Ni, with the highest *ρ*_*i*_/*Z*_*i*_ ratio and the least concentrated around Ti corner in the compositional coordinates, as shown in Fig. 7.

### ToF-SIMS measurements

ToF-SIMS was performed on selected compositions to evaluate the degree of interdiffusion between layers. Blanket film stacks reproducing the stack on the selected locations were prepared for the ToF-SIMS measurement. The ToF-SIMS (ToF-SIMS 5, ION-TOF GmbH) measurements were conducted with a Bi^+^ pulsed primary ion beam (30 keV) for the analysis and O^2+^ beam for sputtering. The non-interlaced mode was used, with 3 s of sputtering before each data point was collected. The analysis area was centred inside the O^2+^ raster area. Because the erosion rate varies with materials quite significantly, even for the same material before and after annealing, the thickness of each layer cannot be determined simply based on the sputtering time unless carefully calibrated.

## Data Availability

The data used to support the findings of this study are included within the article.
